# A Cross-Sectional Study of Pesticide Use and Knowledge of Smallholder Potato Farmers in Uganda

**DOI:** 10.1155/2015/759049

**Published:** 2015-10-25

**Authors:** Joshua Sikhu Okonya, Jürgen Kroschel

**Affiliations:** ^1^DCE Crop Systems Intensification and Climate Change (CSI-CC), International Potato Center (CIP), P.O. Box 22274, Kampala, Uganda; ^2^DCE Crop Systems Intensification and Climate Change (CSI-CC), International Potato Center (CIP), P.O. Box 1558, Lima 12, Peru

## Abstract

In response to increased pest and disease problems, potato farmers use pesticides, which could raise environmental and health concerns. This study sought to promote proper and safe pesticide-handling practices by providing data needed to guide pesticide regulation policy and training for extension staff and farmers. A household survey was conducted in three major potato-growing agroecological zones of Uganda. Two hundred and four potato farmers were interviewed about the type and source of pesticides they use in potato cultivation, the frequency of applications, the use of protective clothing, and cases of pesticide poisoning. The types of pesticides used in potato were fungicides (72%), insecticides (62%), and herbicides (3%). Overall, use of personal protective equipment was low, that is, gumboots (73%), gloves (7%), face masks (16%), and long sleeve shirts (42%). Forty-three percent of farmers who applied pesticides reported having experienced skin itching, 25% skin burning sensation, 43% coughing, 60% a runny nose, 27% teary eyes, and 42% dizziness. An IPM approach involving only moderately to slightly hazardous pesticides when pest and disease incidence has reached economic injury levels and by considering all safety measures during application and storage would be environmentally recommendable and result in reduced health risks.

## 1. Background

Potato (*Solanum tuberosum* L.) is an important food security and cash crop for smallholder farmers in midelevation and highland areas of Uganda with an annual production of 0.8 million tonnes, produced on approximately 112,000 ha [1]. It ranks 5th among the food crops grown in Uganda after sweet potato (*Ipomoea batatas* (L.) Lam.), maize (*Zea mays* L.), cassava (*Manihot esculenta* Crantz), and banana (*Musa* spp.). Most (71%) of the potato produced is for sale as ware potato in local markets with limited formal and informal cross border trade to neighbouring countries of Rwanda and Democratic Republic of Congo [2].

Pests and diseases are among the most important constraints to potato production in Uganda. If not adequately controlled, yield losses from fungal and bacterial diseases alone can reach up to 100% [3]. Yield losses from insect pests in Uganda have not been quantified although their severity and damage is feared to become important with global warming [4, 5]. The absence of environmentally friendly approaches for management of potato pests and diseases has left farmers with no option other than use of chemical pesticides on a routine basis.

Farmers get exposed to toxic pesticides by eating while spraying, entering into freshly sprayed fields, inhalation, and direct contact of the skin with any form (liquid, powder, or aerosol) of pesticides [6]. The Food and Agriculture Organization (FAO) Code of Conduct for Pesticide Use is most of the time not adhered to in many developing countries [7–10]. Misuse of pesticides can lead to illness which reduces the availability of family farm labour and increases the resistance of pests to pesticides due to low pesticide rates and the frequent use of the same active ingredients [11]. In Uganda, the impact of pesticides on human health, environment, and farm productivity among potato farmers has never been estimated. However, isolated cases of farm workers using pesticides to commit suicide do occur. Ngowi et al. [12] observed that it is a challenge to estimate all costs to human health (medical expenses, recuperation costs, transport costs, and labour losses) and the environment (ecosystem degradation) resulting from pesticide use.

Indiscriminate use of pesticides, however, raises a number of environmental and health concerns including soil and water pollution and human and livestock diseases among others. For instance, high pesticide residue levels have been reported in water bodies and foods. Evidence of pesticide poisoning, unsafe pesticide-handling practices, and inadequate use of personal protective equipment has been reported among farmers of horticultural crops in Uganda [13] and coffee (*Coffea arabica* L.) in Jamaica [14]. In 2002, 103 cases of pesticide poisoning leading to four deaths were registered in Poland [15]. However, there are barely any statistics in Uganda for cases of agricultural pesticide poisoning since most farmers are rural and do not seek treatment from hospitals. Even if treatment was sought, it is more likely that health care providers are not adequately trained to make proper diagnosis of pesticide-related illnesses as has been observed in Ghana [9], Ivory Coast [10], Tanzania [16], and South Africa [17]. Some programs such as the Pesticides Initiative Programme that promotes safe pesticide use especially in fresh export produce do exist in Uganda but no such program is known to exist for nonexport produce like potato [18]. The lack of knowledge or training in safe pesticide-handling practices, however, exposes both the environment and potato farmers to the negative effects of pesticides. There is a need to set up policies and programs to promote the safe use of pesticides. Adherence to the international food safety standards will increase not only market avenues of potato but also household income. Integrated Pest Management (IPM) strategies for potato pests ought to be promoted in Uganda to reduce the overall use of pesticides.

This study sought to (i) identify the types of pesticides used in potato farming systems in Uganda, (ii) document the self-reported symptoms of pesticide poisoning, and (iii) describe pesticide-handling practices among potato farming households.

## 2. Materials and Methods

### 2.1. Study Area

Six subcounties (Muko, Nyarusiza, Kapchesombe, Wanale, Kibalinga, and Kakabara) in six major potato-growing districts of Uganda (Kabale, Kisoro, Kapchorwa, Mbale, Mubende, and Kyegegwa), respectively, were purposely selected for this study. District selection was based on representation of the three most important potato-growing agroecological zones of Uganda, that is, southwestern highlands (Kabale and Kisoro), eastern highlands (Mbale and Kapchwora), and Lake Albert Crescent (Mubende and Kyegegwa) districts. One subcounty in each district that was observed by the agricultural extension officers to grow most of the amount of potato was purposively selected. Verbal informed consent was sought from the respondents prior to the beginning of the interview. Respondents were informed of their right to refuse participation and to withdraw from the study at any given time. The confidentiality of the collected information was also assured.

### 2.2. Sampling Procedures

Farm household selection was random and involved stopping at regular intervals (1–5 km) along main roads traversing each subcounty. Respondents were household heads or any adult household member who had grown potatoes in the previous cropping season and was present at home at the time of the study. Two hundred and four potato farmers (34 per district and subcounty) verbally consented to be interviewed. A structured questionnaire was used to interview farmers. The questionnaire was written in English and administered in English and local languages (Luganda, Kupsabiny, Lumasaaba, Rutooro, Rukiga, and Rufumbira) by agriculture extension officers and research assistants under the supervision of the first author.

The interviews covered the following themes: (1) the type and source of pesticides used in potato farming, (2) frequency of pesticide application in a cropping season, (3) the use of protective gear when applying pesticides, (4) any cases of pesticide poisoning experienced by potato farmers, and (5) individual knowledge on the negative effects of pesticide use on the environment among others. Data for this household baseline survey were collected between August and September 2013.

### 2.3. Statistical Analysis

Raw data were coded, entered, and analyzed using the statistical program SAS V.9.2 for Windows (SAS, Cary, NC, USA) [19]. For each agroecological zone, a chi-square test was used to test whether the obtained data and their differences were significant or whether variables were related to each other. The significance levels were set at *P* ≤ 0.01, *P* ≤ 0.05, and *P* ≤ 0.1. The results were then presented in tables separately for each agroecological zone, from which inferences were drawn.

## 3. Results and Discussion

### 3.1. Sociodemographic Profile

Of the 68 respondents that were interviewed per agroecological zone, the number of females and males was not significantly different at *P* ≤ 0.1 for all the three agroecological zones (Table 1). Respondents were mainly between the ages of 31–64 years, followed by the youth (18–30 years). Most of the respondents had attended school for 1–7 years with the Lake Albert agroecological zone having the largest proportion of farmers (72%) in this category.

### 3.2. Pesticide Groups Used by Potato Farmers

All farmers in the southwestern highlands used insecticides and fungicides on potato followed by farmers in the eastern highlands (Table 2). Pesticides were significantly least used in the Lake Albert Crescent with only 16% and 12% of the farmers using fungicides and insecticides, respectively. Generally, herbicides were used by very few farmers (3%) and no farmer in the southwestern highlands used herbicides. The use of both fungicides and insecticides by a large percentage of farmers indicates that fungal diseases specifically late blight and insect pests are perceived to be equally important.

### 3.3. Active Ingredients and Toxicity Classes of Pesticides Used by Potato Farmers

The classification of pesticide active ingredients in this study followed the WHO Recommended Classification of Pesticides by Hazard and Guidelines to Classification 2009 [20]. Most (54.9%) of the fungicides used belonged to the WHO class U (unlikely to present acute hazard in normal use) while 28.9% of the insecticides belonged to the WHO class II (moderately hazardous) (Table 3). Only one highly hazardous (Class 1b) insecticide was used by very few (0.5%) farmers. Due to the lack of formal seed potato suppliers, farmers often save potatoes from the previous own harvest for use as seed in the next cropping season. To control the potato tuber moth* Phthorimaea operculella* (Zeller) during storage, farmers used malathion in southwestern highlands. Some farmers (2.5%) did not know the name of the fungicide they used since it was sold to them in unlabelled polythene bags. Nearly equal number of farmers used fungicides (75.1%) and insecticides (76.5%). However, herbicide use was very low among potato farmers (5.4%).

Highly hazardous pesticides have been reportedly used in many low- and middle-income countries like Peru and Ecuador [8], Philippines [21, 22], Cambodia [23], and Kenya [24]. In Uganda, moderately hazardous pesticides like lambda-cyhalothrin, dimethoate, chlorpyrifos, and cypermethrin have been used in cowpea (*Vigna unguiculata* L. Walp.) [25]. Jensen et al. [23] urged that farmers often think that broad spectrum pesticides are more effective at controlling pests and diseases and therefore the widespread use of highly and moderately hazardous pesticides.

### 3.4. Frequency of Pesticide Application

The number of pesticide applications per season of three months was highest in the eastern highlands for fungicides (5.3 ± 0.4) and insecticides (4.2 ± 0.3) but lowest in Lake Albert Crescent for both fungicides (2.2 ± 0.3) and insecticides (1.4 ± 0.3) (Table 4). Some farmers applied fungicides up to 18 times and insecticides up to 12 times per cropping season.

Frequencies of pesticide application of twice a week have been reported in other crops like tomato (*Lycopersicon esculentum* Mill.) in Uganda [13]. Other countries in Africa reporting heavy use of pesticides include Ghana where tomato farmers sprayed up to 12 times per season [9] and Tanzania where vegetable farmers sprayed up to 16 times per cropping season [12]. Spray frequencies observed in this study are relatively low and may be economical [3].

An Integrated Pest Management approach that has been specifically developed to control economically important potato pests in Uganda involving pesticide applications only when pest and disease incidence has reached economic injury levels would be more sustainable and economically friendly to the environment and hence would also reduce health risks of farmers and consumers. Calendar spraying has also been reported to reduce pests' natural enemies and increase the pest burden [26]. In a related study, we also noted that potato farmers lack general knowledge on the existence of other pest management strategies like the use of intercropping, early planting, early harvesting, use of trapping devices, sanitation, crop rotation, biopesticides, and biological control agents in an Integrated Pest Management approach [27]. IPM for both insect and disease management has to be region specific. IPM for disease (bacterial wilt, viruses, and late blight) management also involves a combination of a number of approaches including use of resistant varieties, clean seed, fungicides, cultural practices (planting at high altitude, crop rotation), and farmer education [28]. In the Andean region of Peru, for instance, IPM for insect management involving the use of plastic barriers, attract-and-kill, and one application of a low-toxic insecticide has been shown to be effective in preventing Andean potato weevils (*Premnotrypes* spp.) infestations, managing potato tuber moths (*Phthorimaea operculella* (Zeller) and* Symmetrischema tangolias* (Gyen)), and controlling flea beetles (*Epitrix* spp.) [29]. In the Republic of Yemen,* P. operculella* was the only economically important potato pest which could be controlled by using healthy uninfested seed and biological control both under field and storage conditions [30, 31]. There is therefore a need to bring to the attention of farmers the existence of more environmentally friendly pest management methods that can increase profit margins.

### 3.5. Sources of Pesticides and Pesticide Information

Most farmers received information about which pesticide to use from other farmers (45%) and only 2% of the farmers received information directly from agricultural extension officers (Table 5). When it came to the doses of pesticides to use, farmers in the southwestern highlands and eastern highlands relied mostly on their own previous experience and reading instructions on the pesticide label (38% and 55%, resp.) while in Lake Albert Crescent, most farmers (50%) relied on pesticide retailers.

On average, agroinput shops were the primary source of pesticides in the three agroecological zones (60%), followed by general household merchandise shops (40%). Other farmers (1%) represented a minor role as source of pesticides. Pesticides were dispensed in quantities of 0.8 ± 0.1 to 8.2 ± 3.5 Kg or litres and it was common to find small quantities of fungicides in unlabelled plastic polythene bags. All farmers used knapsack sprayers to apply pesticides.

### 3.6. Knowledge of Pesticide Toxicity Labels

Less than half of the respondents could read the pesticide labelling across the three agroecological zones; almost all respondents (91%) were not able to explain the toxicity label (Table 6). The relatively low level of education by the majority of the farmers (i.e., <7 years of school) may explain the inability of farmers to read pesticide labels which are often written in English. For the few farmers who knew how to read but did not read the pesticide label, it could be due to reluctance or ignorance of its presence. It should be noted that there is no legislative control in Uganda requiring sellers and users of pesticides to be formally trained. This weakness on the part of the pesticide regulatory bodies may explain the presence and use of highly hazardous (Class 1b) insecticides such as dichlorvos pesticides on the market and the reluctance to use personal protective equipment during pesticide application reported in Table 8.

About a third of the farmers mentioned as negative effects of pesticides symptoms of illness, reduced soil fertility, reduction of beneficial insects, pollution of water sources, and also crop biodiversity loss relating this specifically to the disappearance of the red-fruited nightshade (*Solanum villosum* Miller) in the southwestern highlands. Nearly half (49%) of the farmers in the southwestern agroecological zone applied pesticides before disease symptoms or insect pests occurred. The number of farmers who routinely applied pesticides was lowest in the eastern highlands. There was significant chronic exposure to pesticides among potato farmers (76% and 58% of the farmers in the southwestern and eastern highlands, resp.) of more than 10 years. Majority of farmers (91% in the eastern highlands, 89% in the southwestern highlands, and 67% in Lake Albert Crescent) perceived that the use of pesticides in potato farming has increased in the last 10 years. This trend in pesticide use could be due to increased disease and pest incidence as a result of increased potato production and climate change. It is also possible that the protection against crop loss reaped from calendar spraying has led to high frequencies of pesticide applications in potato [32].

Nearly two-thirds of the farmers applied pesticides in mixtures. It was common for farmers to combine a contact and systemic fungicide plus an insecticide within a single tank mixture to reduce costs for pesticide applications. Reducing costs associated with spraying was also the main reason for combining more than one pesticide among potato farmers in Ecuador [33] and vegetable farmers in Tanzania [12]. Although mixing pesticides can increase efficacy against pests and diseases compared to single applications of each pesticide, care should be taken to ensure that the pesticides being combined are compatible with no antagonism and cannot cause plant toxicity [34].

### 3.7. Farmers' Reports of Pesticide Poisoning Symptoms

Several farmers reported having felt sick after application of pesticides (Table 7). A runny nose was the most common reported symptom by 54%, 72%, and 40% of the farmers in the southwestern highlands, eastern highlands, and Lake Albert Crescent. Skin burning and eye irritation were less common.

Headache, dizziness, itchy skin, cough, dry throat, blurring of vision, general body weakness, and sneezing are some of the most common mild poisoning symptoms usually experienced by pesticide sprayers [10, 12, 23, 35]. Contact with pesticides has been reported to cause higher risk of cancers, neuropsychological impairments, accidental mortality, leukaemia, and even death [15, 22, 36, 37]. Though no cases of deaths were reported in this study, pesticide self-poisoning accounts for about one-third of the world's suicides [38]. During 2002 in Uganda, pesticides accounted for 46% of self-poisoning episodes that received hospital admissions [39]. It should be noted however that even fungicides like mancozeb which are unlikely to cause acute hazard in normal use can lead to long-term risk for cancer development and endocrine disruption [40].

### 3.8. Use of Personal Protective Equipment as Reported by Farmers

Use of personal protective equipment while applying pesticides was very low despite the high risk and frequency of exposure. Boots were the protective equipment worn by majority of the farmers (66%, 83%, and 65% in the southwestern highlands, eastern highlands, and Lake Albert Crescent, resp.), and practically no farmer used a hat, an overall, or goggles (Table 8). Very few farmers used gloves when handling pesticides. Handkerchiefs were often used instead of face and nose masks which likely give a much lower level of protection. The low investment in protective clothing during pesticide handling could be explained by the lack of knowledge on the pesticide toxicity plus the high levels of poverty which makes farmers unable to buy protective clothing.

Relatively very few farmers sought medical treatment after getting signs of pesticide poisoning and the cost of medication was relatively low (≤2$US, data not shown), not considering overall costs which would include consultation fees, cost of diagnosis, travel to and from the health centres, cost of time spent in the health centre, and costs out of productive work, among others.

Farmers often believe that pesticide-related symptoms are normal and therefore do not seek medical treatment as was the case in Tanzania [12], Indonesia [41], and Ivory Coast [10].

## 4. Conclusions, Recommendations, and Policy Implications

This baseline study gives an insight into the range of pesticides used in the management of potato pests and diseases in Uganda, pesticide-handling practices, and symptoms of occupational pesticide poisoning. The protection against loss reaped from calendar spraying has led to high frequencies of pesticides applications in potato cultivation. Many farmers in the study areas are not adequately informed about the hazards associated with pesticide use and do not strictly use protective measures to guard them and the environment from hazards of pesticide exposure. The improper use of highly and moderately hazardous pesticides by farmers often resulted in pesticide poisoning among farmers. However, the affected farmers rarely sought medical treatment. More in-depth studies on the impact of pesticide exposure on the livelihoods are recommended. Information gathered in this study will help to guide or improve future pesticide regulation and health interventions.

The lack of knowledge of pesticide use and handling calls for investments in farmer training by governmental extension organizations, NGOs, pesticides policy and regulatory bodies, food safety standard regulatory organization(s), and the Ministry of Agriculture. An Integrated Pest Management approach would be the most effective way of reducing pesticide use in potato production while protecting the environment, increasing the productivity of potato, promoting natural enemy population build-up, and reducing the development of pesticide resistance and human health related risks. This baseline survey is the first step towards the development of an IPM system conducive to Ugandan potato farming systems.

## Figures and Tables

**Table 1:** Demographic characteristics of potato farmers interviewed in August and September 2013.

Demographic variable of respondents	Entire sample (mean) *N* = 204	Percent		
		Agroecological zone		
		SWH (*n* = 68)	EH (*n* = 68)	LAC (*n* = 68)
Sex of the respondent				
Male	58	56	56	63
Female	42	44	44	37
Age group				
18–30 years		25	29	30
31–64 years		66	68	66
≥65 years		9	3	4
Sample (mean years ± SE)^*∗*^	39.6 ± 0.9	41.26 ± 1.6^a^	39.28 ± 1.4^a^	38.16 ± 1.5^a^
Education level				
None (0 years)		18	7	15
Primary (1–7 years)		56	52	72
Secondary (8–11 years)		13	30	10
Advanced secondary (12-13 years)		3	9	3
University/college (≥14 years)		10	1	0
Sample (mean years ± SE)^*∗*^	6.0 ± 0.3	6.24 ± 0.6^a^	7.12 ± 0.5^a^	4.73 ± 0.4^b^

^*∗*^Mean values with the same letter are not significantly different at *P* ≤ 0.05. SWH: southwestern highlands; EH: eastern highlands; LAC: Lake Albert Crescent. Numbers of female and male respondents were not significantly different at *P* ≤ 0.1 for all the three agroecological zones.

**Table 2:** Percentage of potato farmers using each group of pesticides by agroecological zone in Uganda.

Percentage of farmers using each pesticide group	Entire sample mean (*n* = 204)	Agroecological zone			Chi^2^		
		SWH (*n* = 68)	EH (*n* = 68)	LAC (*n* = 68)	SWH versus EH	SWH versus LAC	EH versus LAC
(1) Fungicides	72	100	99	16	1.01^ns^	98.13^*∗∗∗*^	94.27^*∗∗∗*^
(2) Insecticides	62	100	75	12	19.43^*∗∗∗*^	107.37^*∗∗∗*^	55.35^*∗∗∗*^
(3) Herbicides	3	0	1	9	1.01^ns^	6.28^*∗∗*^	3.77^*∗*^

*∗∗∗*, *∗∗*, and *∗* indicate statistical significance at *P* ≤ 0.01, *P* ≤ 0.05, and *P* ≤ 0.1, respectively. ns: not statistically different at *P* ≤ 0.1. *n* = number of respondents. SWH: southwestern highlands; EH: eastern highlands; LAC = Lake Albert Crescent.

**Table 3:** Commercial names, active ingredients, and WHO toxicity classes of pesticides used by potato farmers in Uganda.

Number	Commercial pesticide name(s)	Active ingredient(s) and concentration	Reported use (% responses)^*∗*^	*WHO toxicity class* ^(a)^
(a) Fungicides (*n* = 146)				
1	Tata master 56	Mancozeb 48% + Metalaxyl 10%	9.8	II
2	Agro-Laxyl MZ 63.5 WP	Mancozeb 56% + Metalaxyl 7.5%	2.0	II
3	Ridomil Gold MZ 68 WG	Mancozeb 64% + Metalaxyl 4%	5.4	II
4	Orius 25 EC	Tebuconazole 25%	0.5	II
5	Dithane M45	Mancozeb 80%	54.4	U
5	Greenzeb 80 WP	Mancozeb 80%		U
5	Indofil M45	Mancozeb 80%		U
5	Agrozeb 80 WP	Mancozeb 80%		U
5	Greenzeb 80 WP	Mancozeb 80%		U
5	Mancozeb 80% WP	Mancozeb 80%		U
6	Antracol 70 WP	Propineb 70%	0.5	U
7	Unknown		2.5	—
	Total responses (fungicides)		**75.1**	

(b) Insecticide (*n* = 127)				
1	Lava 100% EC	Dichlorvos 100%	0.5	Ib
2	Bulldock 0.25 EC	Beta-cyfluthrin 2.5%	0.5	II
3	Ambush	Permethrin 50% EC	30.9	II
4	Rocket 44 EC	Cypermethrin 4% + Profenofos 40%	14.2	II
5	Dudu Ethoate	Dimethoate 40%	7.8	II
5	Super Ethoate	Dimethoate 40%		II
5	Agrithoate 40 EC	Dimethoate 40%		II
5	Tafgor 40 EC	Dimethoate 40%		II
6	Ambush Super	Lambda-cyhalothrin	0.5	II
7	Cyclone 505 EC	Cypermethrin 10% + chlorpyrifos 35%	0.5	II
8	Dudu Alpha	Alpha-cypermethrin 3%	1.0	II
9	Dudu Cyper	Cypermethrin 5%	4.4	II
	CyperLacer 5 EC	Cypermethrin 5%		II
10	Dursban 48 EC	Chlorpyrifos 48%	0.5	II
11	Malataf 57 EC	Malathion 57%	15.7	III
	Total responses (insecticides)		**76.5**	

		(c) Herbicides (*n* = 6)		
1	Green-2,4-D	2,4-D-Amine 860 g/L	0.5	II
2	Roundup 36% SL	Glyphosate 36%	2.5	III
2	Mamba	Glyphosate 36%		III
3	Green Fire 50% SL	Glyphosate 50%	2.5	III
3	WeedMaster 50% SL	Glyphosate 50%		III
	Total responses (herbicides)^*∗*^		**5.5**	

	Grand Total responses (pesticides)^*∗*^, *n* = 279		**157.1**	

^*∗*^Multiple responses; *n* = number of responses; ^(a)^Ib: highly hazardous; II: moderately hazardous; III: slightly hazardous; U: unlikely to present acute hazard in normal use.

**Table 4:** Number (mean ± SE) of pesticide applications per season by potato farmers in Uganda.

Number of pesticide spray regimes per season of three months	Entire sample mean ± SE (*n* = 204)	Agroecological zone (mean ± SE)		
		SWH (*n* = 68)	EH (*n* = 68)	LAC (*n* = 68)
(1) Fungicides	4.5 ± 0.2	4.2 ± 0.2^a^	5.3 ± 0.4^a^	2.2 ± 0.3^b^
(2) Insecticides	3.8 ± 0.2	3.7 ± 0.1^a^	4.2 ± 0.3^a^	1.4 ± 0.3^b^
(3) Herbicides	1.0 ± 0.0	0.0 ± 0.0	1.0 ± 0.0^a^	1.0 ± 0.0^a^

Mean values with the same letter in the same row are not significantly different at *P* ≤ 0.05. SWH: southwestern highlands; EH: eastern highlands; LAC: Lake Albert Crescent.

**Table 5:** Sources of pesticides and pesticide information for potato farmers in Uganda.

	Entire sample (mean)	Agroecological zone			Chi^2^		
		SWH	EH	LAC	SWH versus EH	SWH versus LAC	EH versus LAC
Percentage of farmers who received information about choice of a pesticide to buy or apply	*n* = 154	*n* = 68	*n* = 67	*n* = 19			

(1) Agrochemical retailers	34	29	36	42	5.47^ns^	1.81^ns^	1.11^ns^
(2) Other farmers	45	54	37	42			
(3) Previous experience	19	13	25	16			
(4) Agriculture extension officers	2	3	1	0			

Percentage of farmers who received information about pesticide doses	*n* = 155	*n* = 68	*n* = 67	*n* = 20			

(1) Agrochemical retailers	33	26	34	50	12.52^*∗∗*^	4.50^ns^	4.09^ns^
(2) Other farmers	21	31	10	20			
(3) Previous experience/can read	45	38	55	30			
(4) Agriculture extension officers	1	3	0	0			
(5) Agrochemical retailers + agriculture extension officers	1	1	0	0			

Place where pesticides were bought	*n* = 156	*n* = 68	*n* = 68	*n* = 20			

(1) Agrochemical retailers	60	35	90	40	43.03^*∗∗∗*^	0.41^ns^	22.55^*∗∗∗*^
(2) General household merchandise shops	40	63	10	60			
(3) Other farmers	1	1	0	0			

*∗∗∗*, *∗∗*, and *∗* indicate statistical significance at *P* ≤ 0.01, *P* ≤ 0.05, and *P* ≤ 0.1, respectively. ns: not statistically different at *P* ≤ 0.1. SWH: southwestern highlands; EH: eastern highlands; LAC: Lake Albert Crescent.

**Table 6:** Knowledge and attitudes towards pesticides among potato farmers in Uganda.

Percent pesticide use practices (*n*)	Entire sample (mean) *N* = 155	Agroecological zone			Chi^2^		
		SWH	EH	LAC	SWH versus EH	SWH versus LAC	EH versus LAC
(1) Can read and understand the pesticides labels	38 (154)	29 (68)	46 (67)	35 (20)	4.08^*∗∗*^	0.23^ns^	0.80^ns^

(2) Aware of the toxicity color codes present on the pesticide containers	9 (152)	4 (68)	9 (65)	25 (20)	1.22^ns^	7.93^*∗∗∗*^	3.38^*∗*^

(3) Aware of the negative effects of pesticides on the environment and health	35 (154)	31 (68)	39 (67)	37 (19)	0.93^ns^	0.24^ns^	0.02^ns^

(4) Applies pesticides on a routine basis	35 (154)	49 (68)	21 (67)	40 (20)	11.36^*∗∗∗*^	0.45^ns^	2.98^*∗*^

(5) Has been using pesticides on potato for >10 years	59 (155)	76 (68)	58 (67)	5 (20)	5.12^*∗∗*^	32.96^*∗∗∗*^	17.56^*∗∗∗*^

(6) Pesticides use in potato has increased in the last 10 years	89 (107)	89 (56)	91 (45)	67 (6)	0.09^ns^	2.47^ns^	3.05^*∗*^

(7) Used tank mixtures of different pesticides on potato	64 (154)	91 (68)	51 (67)	11 (19)	26.86^*∗∗∗*^	49.67^*∗∗∗*^	9.13^*∗∗∗*^

(8) Sex of pesticide sprayer in a household (F: female, M: male)	F = 6 M = 87 M and F = 6 (154)	F = 3 M = 90 M and F = 7 (68)	F = 10 M = 82 M and F = 7 (67)	F = 5 M = 95 (19)	3.08^ns^	1.67^ns^	2.12^ns^

*∗∗∗*, *∗∗*, and *∗* indicate statistical significance at *P* ≤ 0.01, *P* ≤ 0.05, and *P* ≤ 0.1, respectively. ns: not statistically different at *P* ≤ 0.1. SWH: southwestern highlands; EH: eastern highlands; LAC: Lake Albert Crescent. The sample size (*n*) for each percentage is indicated in parenthesis.

**Table 7:** Effects of pesticide exposure reported by farmers during and after pesticide application in Uganda.

Symptoms	Entire sample (mean) *N* = 154	Agroecological zone			Chi^2^		
		SWH (*n* = 68)	EH (*n* = 67)	LAC (*n* = 20)	SWH versus EH	SWH versus LAC	EH versus LAC
(1) Itchy skin	43	28	61	35	15.11^*∗∗∗*^	0.37^ns^	3.56^*∗*^
(2) Skin burning sensation	25	22	27	30	0.42^ns^	0.54^ns^	0.16^ns^
(3) Runny nose	60	54	72	40	4.30^*∗∗*^	1.28^ns^	7.78^*∗∗∗*^
(4) Coughing	43	31	61	20	12.49^*∗∗∗*^	0.90^ns^	12.21^*∗∗∗*^
(5) Teary eyes/eye irritation	27	12	42	25	15.56^*∗∗∗*^	2.15^ns^	1.84^ns^
(6) Dizziness/headache	42	46	42	25	0.20^ns^	2.71^ns^	2.72^*∗*^

*∗∗∗*, *∗∗*, and *∗* indicate statistical significance at *P* ≤ 0.01, *P* ≤ 0.05, and *P* ≤ 0.1, respectively. ns: not statistically different at *P* ≤ 0.1. SWH: southwestern highlands; EH: eastern highlands; LAC: Lake Albert Crescent.

**Table 8:** Use of protective clothing during pesticide application by potato farmers in Uganda (% responses).

Personal protective equipment	Entire sample (mean) *N* = 154	Agroecological zone			Chi^2^		
		SWH (*n* = 68)	EH (*n* = 66)	LAC (*n* = 20)	SWH versus EH	SWH versus LAC	EH versus LAC
(1) Gloves	7	3	12	5	3.89^*∗∗*^	0.20^ns^	0.80^ns^
(2) Boots	73	66	82	65	3.94^*∗∗*^	0.01^ns^	2.64^ns^
(3) Face and nose mask	16	10	19	20	2.22^ns^	1.33^ns^	0.00^ns^
(4) Long sleeved shirt and trousers	42	25	60	35	16.15^*∗∗∗*^	0.78^ns^	3.78^*∗*^

*∗∗∗*, *∗∗*, and *∗* indicate statistical significance at *P* ≤ 0.01, *P* ≤ 0.05, and *P* ≤ 0.1, respectively. ns: not statistically different at *P* ≤ 0.1. SWH: southwestern highlands; EH: eastern highlands; LAC: Lake Albert Crescent.

## Abbreviations

CIP: International Potato Center
CSI-CC: Crop Systems Intensification and Climate Change
DCE: Disciplinary Center of Excellence
FAO: The Food and Agriculture Organization
IPM: Integrated Pest Management
NGO: Nongovernmental Organization
WHO: The World Health Organization.
